# Evaluation of an Adsorbent Based on Agricultural Waste (Corn Cobs) for Removal of Tyrosine and Phenylalanine from Aqueous Solutions

**DOI:** 10.1155/2013/978256

**Published:** 2013-07-02

**Authors:** Cibele C. O. Alves, Adriana S. Franca, Leandro S. Oliveira

**Affiliations:** ^1^PPGCA/Universidade Federal de Minas Gerais, Avenida Antonio Carlos 6627, 31270-901 Belo Horizonte, MG, Brazil; ^2^DEMEC/Universidade Federal de Minas Gerais, Avenida Antônio Carlos 6627, 31270-901 Belo Horizonte, MG, Brazil

## Abstract

Adsorption of phenolic amino acids, such as phenylalanine and tyrosine, is quite relevant for the production of protein hydrolysates used as dietary formulations for patients suffering from congenital disorders of amino acid metabolism, such as phenylketonuria. In this study, an adsorbent prepared from corn cobs was evaluated for the removal of tyrosine (Tyr) from both a single component solution and a binary aqueous solution with phenylalanine (Phe). The adsorption behavior of tyrosine was similar to that of phenylalanine in single component solutions, however, with a much lower adsorption capacity (14 mg g^−1^ for Tyr compared to 109 mg g^−1^ for Phe). Tyr adsorption kinetics was satisfactorily described by a pseudosecond-order model as it was for Phe. In adsorption equilibrium studies for binary mixtures, the presence of Tyr in Phe solutions favored Phe faster adsorption whereas the opposite behavior was observed for the presence of Phe in Tyr solutions. Such results indicate that, in binary systems, Phe will be adsorbed preferably to Tyr, and this is a welcome feature when employing the prepared adsorbent for the removal of Phe from protein hydrolysates to be used in dietary formulations for phenylketonuria treatment.

## 1. Introduction

Phenylketonuria (PKU) is the most common inborn error of amino acid metabolism, being due to a deficiency of the enzyme phenylalanine hydroxylase, which normally converts phenylalanine (Phe) to tyrosine (Tyr) [1]. PKU can result in several problems in untreated patients, including intellectual disability, liver failure, and reduction of life expectancy [1, 2], and its nutritional therapy is accomplished by rigorous diets based on Phe-free protein substitutes, either mixtures of free amino acids or protein hydrolysates [3]. In Brazil, the mixtures of free amino acids are imported and available at high costs. One alternative to reduce costs is the use of residues from the food industry in the development of protein hydrolysates. As an example, some studies have shown that this can be accomplished with whey protein, a residue from milk and cheese production [4, 5]. However, the produced hydrolysates still contain Phe, and its contents must be reduced to acceptable levels, usually by adsorption with activated carbons or synthetic adsorbents [4, 6, 7]. High costs are still associated with production or regeneration of such adsorbents, and these costs could be further reduced by the use of low-cost adsorbents based on agricultural wastes [8].

**Figure 1:**
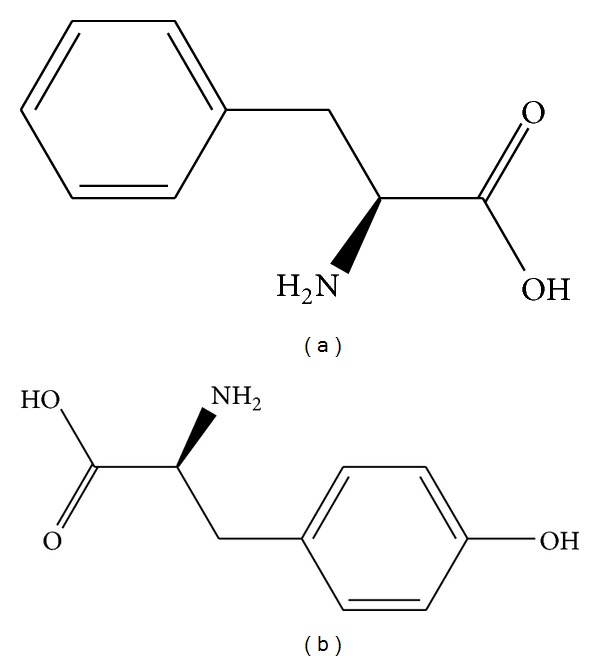
Chemical structure of (a) phenylalanine-Phe and (b) tyrosine-Tyr.

Reports on the use of agricultural residues for removal of aminoacids from aqueous solutions by adsorbents based on agricultural residues are not available in the literature, with the exception of our previous studies on removal of Phe from aqueous solutions [9, 10]. We have evaluated the feasibility of employing defective coffee bean press cake [9] and corn cobs [10] for the production of adsorbents and their further use for phenylalanine adsorption. In both studies, adsorption kinetics was satisfactorily described by a pseudosecond-order model, with Langmuir model providing a satisfactory description of adsorption isotherms. Also, an increase in temperature led to a decrease in the amount adsorbed, indicating that Phe adsorption was exotermic, regardless of the residue employed for adsorbent production. However, even though the activation procedure was the same for both adsorbents, the corn cob-based adsorbent presented significantly higher adsorption capacity (109 mg g^−1^) than the coffee residue-based adsorbent (69.5 mg g^−1^). This adsorption capacity was either equivalent or higher than that of nonresidue-based adsorbents such as zeolites and synthetic resins, confirming the potential of corn cobs as raw materials for production of adsorbents aiming at Phe removal. Nonetheless, one of the major problems in the production of Phe-free hydrolysates is the fact that aromatic aminoacids including both Phe and Tyr (see Figure 1) will be preferably adsorbed with respect to nonaromatic ones [11], and thus there might be a need to reinstate Tyr in the formulation, in order to guarantee an adequate protein quality for the Phe-free diet [12]. In view of the aforementioned, the objective of this work was to evaluate the performance of the previously developed corn cob-based adsorbent [10] for the removal of Tyr in comparison to Phe. We initially evaluated adsorption of pure Tyr, in order to ascertain if the produced adsorbent would be able to remove this aminoacid under ideal conditions. Sequentially, we evaluated binary adsorption, for example, Phe removal in the presence of Tyr and Tyr removal in the presence of Phe, in order to verify the selectivity of the produced adsorbent.

## 2. Materials and Methods

### 2.1. Materials

 Corn cobs were provided by EMBRAPA (Sete Lagoas, Brazil). Phenylalanine and tyrosine standards as well as other reagents were purchased from Sigma-Aldrich (SP, Brazil). The adsorbent was prepared according to the procedure employed in a previous study: 3 min impregnation with H_3_PO_4_ followed by 1 h activation in a muffle furnace at 400°C [10].

### 2.2. Adsorbent Characterization

 Functional groups present at the adsorbent surface were examined using Fourier transform infrared (FTIR) spectroscopy. The FTIR spectra were recorded on a Shimadzu IRAffinity-1 spectrometer (Japan) operating in the range of 400–4000 cm^−1^, with a resolution of 4 cm^−1^. 

### 2.3. Adsorption Studies

Batch experiments of adsorption were performed using 250 mL Erlenmeyer flasks agitated on a shaker (Nova Ética, SP, Brazil) at 100 rpm for predetermined time intervals (5, 15, 30, 60, 180, 360, and 540 min) and the following values of initial Tyr concentration: 20, 30, 50, 75, and 100 mg L^−1^. Adsorption conditions were established as the most adequate for Phe removal, that is, initial solution pH of 6 and adsorbent concentration of 10 g L^−1^, as determined in our previous study [10]. For evaluation of the binary systems, batch adsorption tests were conducted employing equal volumes of each aminoacid solution at the following concentrations: (a) Tyr removal: Phe 500 mg·L^−1^; Tyr ranging from 20 to 150 mg·L^−1^; (b) Phe removal: Tyr 50 mg·L^−1^; Phe ranging from 200 to 1500 mg·L^−1^. Lower concentrations of Tyr in comparison to Phe are due to the fact that the water solubility constant of Tyr is ten times smaller than the one for Phe. After specified time periods, 2 mL aliquots were taken from the Erlenmeyer flasks and Phe and Tyr concentrations were determined by a UV-Vis spectrophotometer (Hitachi U-2010) at 257 and 273 nm, respectively. The amount of aminoacid adsorbed per unit mass of adsorbent, *q*_*t*_ (mg g^−1^), was calculated as

(1) $$\begin{array}{c} q_{t}=\frac{\left(C_{0}-C_{t}\right)V}{W}, \end{array}$$

where *C*_0_ and *C*_*t*_ (mg L^−1^) correspond to the liquid-phase concentrations of Phe or Tyr at initial and sampling times, respectively, *V* is the volume of the solution (L), and *W* is the mass of dry adsorbent used (g). All tests were performed in three replicates. Kinetics and equilibrium studies were performed at 25, 35, and 45°C for pure Tyr, and equilibrium was evaluated at 25°C for the binary system.

## 3. Results and Discussion

### 3.1. Adsorbent Characterization

**Figure 2:**
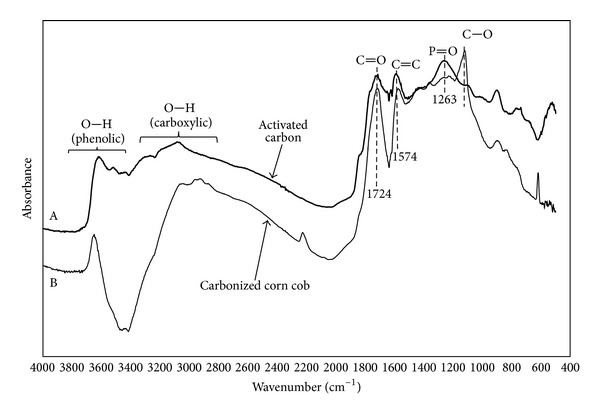
FTIR spectra obtained for the produced activated carbon (A) in comparison to carbonized corn cobs (B).

The FTIR spectrum obtained for the prepared activated carbon is presented in Figure 2(A) in comparison to the spectrum obtained for carbonized corn cobs (B). The spectrum of the activated carbon (A) is similar to others shown in the literature for chemical activation of lignocellulosic materials by H_3_PO_4_ [13, 14]. The bands seen in the region between 1300 and 1000 cm^−1^, with maxima at 1263 (A) and 1121 (B) cm^−1^, are attributed to P=O stretching vibrations (A) and C–O stretching in acids, alcohols, phenols, ethers, and esters (B). The significant difference between these bands confirms the effect of the activation procedure [14]. A small band at 1100 cm^−1^, (A) is attributed to ionized linkage P^+^–O^−^ in phosphate esters or to symmetrical vibration in a P–O–P chain. This band is not present in the spectrum for carbonized corn cob without chemical activation (B) and has been reported to become better defined with an increase in impregnation rate [14]. Bands at 1121 cm^−1^ (–OCH_3_) and near 1724 cm^−1^ (C=O stretching band) have been reported in association with the presence of lignin and hemicellulose esters [15]. Notice that these bands do not appear and/or are less evident in the activated carbon (A) in comparison to the carbonized corn cob (B), probably in association to hydrolysis of lignin and hemicellulose constituent esters by the activating agent. The broad bands at 3200–2700 and 3800–3400 cm^−1^ are attributed to hydrogen bonds in carboxilic and phenolic groups, respectively. Reduction in intensity of such bands after activation could be associated to interactions between the O–H group and the activating agent. A more detailed discussion on the characterization of this adsorbent is available in a previous study [10].

### 3.2. Effects of Initial Tyr Concentration and Contact Time

**Figure 3:**
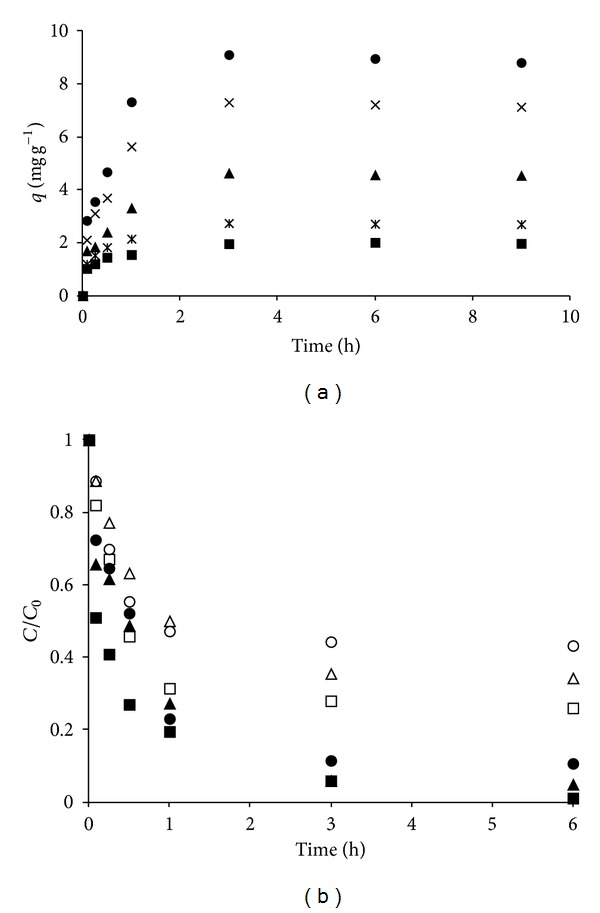
Effect of contact time on Tyr adsorption by the prepared adsorbent: (a) variations in amount adsorbed and (b) variations in final concentration in comparison to Phe removal [9] (25°C; initial solution pH: 6; adsorbent dosage: 10 g·L^−1^; initial Tyr concentration: ■: 20 mg L^−1^; ∗: 30 mg L^−1^; ▲: 50 mg L^−1^;  ×75 mg L^−1^; ●: 100 mg L^−1^. Initial Phe concentration: □: 200 mg L^−1^; △: 500 mg L^−1^; ◯: 1000 mg L^−1^).

The data presented in Figure 3 show that adsorption capacity is directly affected by Tyr initial concentration. As expected, an increase in Tyr initial concentration resulted in an increase in the total amount adsorbed, given the corresponding increase in driving force (concentration gradient). Regardless of the initial Tyr concentration, a two-stage kinetic behavior can be clearly seen, with a rapid initial adsorption during the first hour and a slower rate afterwards. The same qualitative behavior was observed for Phe removal by the same adsorbent [10]. Results in Figure 3(a) also show that a contact time of 3 hours assured attainment of equilibrium conditions for all evaluated initial Tyr concentrations. The same qualitative behavior was observed for experiments conducted at higher temperatures (35 and 45°C) and also for Phe removal (Figure 3(b)). It can be noticed from Figure 3(b) that the differences in the curves are mainly due to the differences in aminoacid initial concentration and not to the aminoacid itself.

### 3.3. Adsorption Kinetics

The controlling mechanism of Tyr removal was investigated by fitting pseudofirst- and second-order kinetic models to experimental data [17], with the kinetic rate equations being represented by

(2) $$\begin{array}{c} \frac{dq_{t}}{dt}=k_{n}\;{\left(q_{e}-q_{t}\right)}^{n}, \end{array}$$

where *q*_*e*_ and *q*_*t*_ correspond to the amount of Tyr adsorbed per unit mass of adsorbent (mg g^−1^) at equilibrium and at time *t*, respectively, and *k*_*n*_ is the rate constant for *n*th-order adsorption (units are h^−1^ for *n* = 1 and g mg^−1^ h^−1^ for *n* = 2). The corresponding integrated equations for the pseudofirst-order (*n* = 1) and pseudosecond-order (*n* = 2) models are

(3) $$\begin{array}{c} q_{t}=q_{e}\left(1-e^{-k_{1}t}\right),\\ q_{t}=\frac{k_{2}q_{e}^{2}t}{1+k_{2}q_{e}t}. \end{array}$$

Evaluation of model ability to predict the experimental data was based on both regression correlation coefficient values (*r* ^2^) and difference between experimental (*q*_*t*,exp⁡_) and model-estimated (*q*_*t*,est_) values, evaluated by means of the error measure:

(4) $$\begin{array}{c} \text{RMS}\left(\%\right)=\frac{100\sqrt{\sum {\left[\left(q_{t,\text{est}}-q_{t,\exp}\right)/q_{t,\exp}\right]}^{2}}}{N}, \end{array}$$

where *N* is the number of experimental points in each *q*_*t*_ versus *t* curve.

The results obtained after nonlinear regression of kinetic models and the corresponding estimates for equilibrium adsorption capacity are presented in Table 1. The pseudosecond order-model provided higher *r* ^2^ values and lower values of RMS error in comparison to the pseudofirst-order model, thus is being considered more adequate for the description of the adsorption data, for all evaluated temperatures. This model has been successfully applied for the description of adsorption kinetics of a variety of adsorbates and adsorbents [17], and it was also the more adequate model for the description of Phe removal from aqueous solutions in our previous studies [9, 10].

### 3.4. Adsorption Equilibrium 

#### 3.4.1. Pure Tyrosine

**Figure 4:**
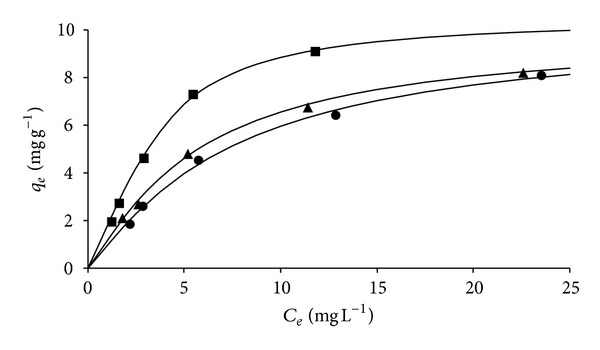
Adsorption isotherms obtained for Tyr removal (■: 25°C; ▲: 35°C; ●: 45°C); solid lines represent Langmuir-Freundlich fits.

The adsorption isotherms for removal of pure Tyr at 25, 35, and 45°C are displayed in Figure 4. The shapes of the curves are characteristic of favorable adsorption, regardless of temperature being similar to the curves obtained for Phe removal [10]. An evaluation of isotherms presented in Figure 4 indicates that Tyr removal is an exothermic process, since increases in temperature led to decreases in the amount adsorbed, as previously reported for Phe [10]. Such behavior can be attributed to the hydrophobic nature of aromatic aminoacids such as Tyr and Phe. As the temperature increases, these aminoacids present greater tendency to form hydrophobic bonds in solution as opposed to interacting with the adsorbent surface [18]. 

Two- and three-parameter models were evaluated for equilibrium description. Model equations and results obtained after nonlinear regression analysis are displayed in Table 2. Model selection was based on the highest *r* ^2^ values coupled with the lowest difference between calculated and experimental results, *q*_*e*_ values, evaluated according to

(5) $$\begin{array}{c} \text{RMS}\left(\%\right)=\frac{100\sqrt{\sum {\left[\left(q_{e,\text{est}}-q_{e,\exp}\right)/q_{e,\exp}\right]}^{2}}}{N}, \end{array}$$

where *q*_*e*,exp⁡_ and *q*_*e*,est_ are the experimental and calculated equilibrium adsorbed amounts, respectively, and *N* is the number of experimental isotherm points.

An evaluation of both *r* ^2^ and RMS values show that Tyr adsorption was better described by the three-parameter Langmuir-Freundlich model, regardless of temperature. Both Langmuir and Temkin models provided a better description than Freundlich, which can be attributed to the microporous nature of the adsorbent [10, 19]. Freundlich equation is an empirical model that does not account for adsorbent saturation and has been associated to both heterogeneous and multilayer adsorption, whereas both Langmuir and Temkin models are based on theoretical approaches assuming monolayer adsorption over an energetically and structurally homogeneous adsorbent surface [20]. Although the best fit was provided by the Langmuir-Freundlich model, for example, a combination of Langmuir and Freundlich isotherms, estimated values of the parameter “*n*” show a tendency to approach one with the increase in temperature (see Table 2), indicating a possible shift from multilayer (Freundlich) to monolayer (Langmuir) adsorption depending on the temperature. Maximum Tyr uptake capacity, based on Langmuir model, was 14 mg g^−1^. This value is low compared to the results reported in the literature [7] for Tyr removal by ion-exchange resins (~54 mg g^−1^). However, the corncob-based adsorbent presented better performance in terms of Phe removal, with maximum uptake capacity of 109 mg g^−1^ [10] compared to 66 mg g^−1^ Phe uptake by ion-exchange resins [7]. Recall that Phe is the target adsorbate in the production of protein hydrolisates, and thus its higher adsorption capacity is welcome.

#### 3.4.2. Binary Adsorption

**Figure 5:**
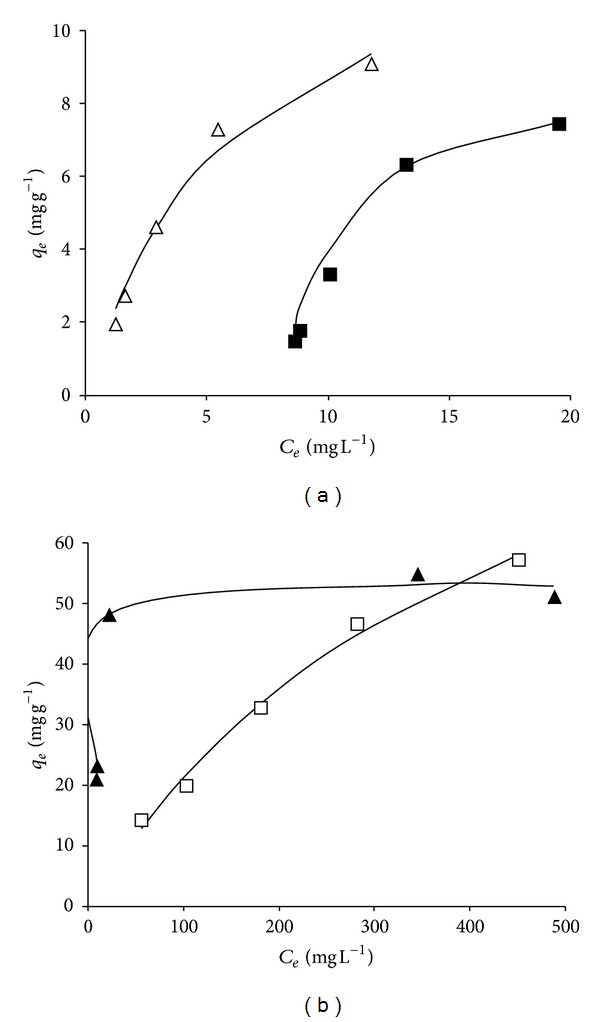
Comparative evaluation of adsorption isotherms for (a) Tyr and (b) Phe at 25°C (■: pure Tyr; △: Tyr in binary solution; □: pure Phe; ▲: Phe in binary solution; solid lines correspond to Langmuir fits for single and binary systems).

The adsorption isotherms obtained at 25°C for each aminoacid in single and binary systems are shown in Figure 5. The shapes of all curves indicate favorable adsorption, for both aminoacids either pure or mixed. A comparison of the curves obtained for single and binary adsorption of each aminoacid shows that, in the case of Phe, the presence of Tyr favored its faster adsorption whereas the opposite behavior was observed for Tyr. Such results indicate that, in the binary systems, Phe will be adsorbed preferably in comparison to Tyr. The adsorption dynamics of the aminoacid mixture can be also evaluated by comparing *qe*′/*qe* ratios [21], where the prime denotes the presence of another aminoacid. In general, three possible types of behavior are exhibited: *qe*′/*qe* > 1, indicating synergism (the effect of the mixture is greater than that of the individual adsorbates in the mixture); *qe*′/*qe* < 1, corresponding to antagonism (the effect of the mixture is less than that of each of the individual adsorbates in the mixture); and *qe*′/*qe* = 1, noninteraction (the mixture has no effect on the adsorption of each of the adsorbates in the mixture). The average value of *qe*′/*qe* ratio was 1.23 for Phe adsorption in the presence of Tyr, suggesting a synergistic effect. However, the average *qe*′/*qe* ratio for Tyr adsorption in the presence of Phe was 0.8, indicating that Tyr adsorption was depressed by Phe in the binary solution. 

Although there are many models for description of single component adsorption isotherms in the literature, the most widely employed are Freundlich and Langmuir [20]. Thus, there have been several previous efforts in the literature with the objective of extending these isotherms to multicomponent systems [22, 23]. The corresponding equations are:

Langmuir:

(6) $$\begin{array}{c} q_{e,i}=q_{m,i}\frac{K_{L,i}C_{e,i}}{1+\sum_{i=1}^{N}K_{L,i}C_{e,i}}. \end{array}$$

Freundlich:

(7) $$\begin{array}{c} q_{e,i}=q_{m,i}{\left[\frac{K_{F,i}C_{e,i}}{1+\sum_{i=1}^{N}K_{F,i}C_{e,i}}\right]}^{y};\;\left(0<y\leq 1\right), \end{array}$$

where *q*_*m*,*i*_ represents the saturation or maximum adsorption capacity of component *i*, *q*_*e*,*i*_ (mg g^−1^) is the equilibrium adsorption capacity of component *i*, *C*_*e*,*i*_ (mg L^−1^) is the concentration of component *i* in the aqueous solution after equilibrium, *K*_*Li*_, *K*_*F*,*i*_ and *y* are the remaining empirical parameters associated to Langmuir and Freundlich models, and *N* is the number of components (2 for binary adsorption). 

The results obtained after nonlinear regression analysis of the binary adsorption isotherms are shown in Table 3. Model selection was based on the highest *r* ^2^ values coupled with the lowest difference between calculated and experimental results, *q*_*e*_ values, evaluated according to

(8) RMS(%)  =12[∑i=12(100∑[(qe,i,est−qe,i,exp⁡)/qe,i,exp⁡]2N)],

where *q*_*e*,*i*,exp⁡_ and *q*_*e*,*i*,est_ are the experimental and estimated equilibrium adsorbed amounts for component *i*, respectively, and *N* is the number of experimental isotherm points. An evaluation of both *r* ^2^ and RMS values indicates that both models provided a satisfactory description of binary adsorption. However, Langmuir provided a slightly lower RMS value for description of Phe removal in the presence of Tyr, whereas the opposite was observed for Tyr removal. Such behavior could be an indication that Phe molecules will probably form a monolayer over the adsorbent surface whereas Tyr molecules will adsorb in multilayers, probably interacting with the adsorbed Phe molecules. This can be corroborated by evaluation of the ratio between the constants of the Langmuir model, *K*_*L*_, that can be associated to the ratio between adsorption and desorption constants during equilibrium. The higher values observed for Phe removal as opposed to Tyr removal (see *K*_*L*1_/*K*_*L*2_ ratios in Table 3) confirm the higher affinity of the adsorbent for Phe.

It is interesting to notice that in general the adsorption behavior observed for pure Tyr was the same observed for pure Phe removal by the same adsorbent [10]. This was expected, given the similarity in molecular structure of both aminoacids and in principle could be an indication that both molecules would compete for adsorption sites. However, the results from binary adsorption indicate that the prepared adsorbent will preferably remove Phe over Tyr, thus, being appropriate for Phe removal in mixtures with other aminoacids.

### 3.5. Adsorption Mechanism

**Figure 6:**
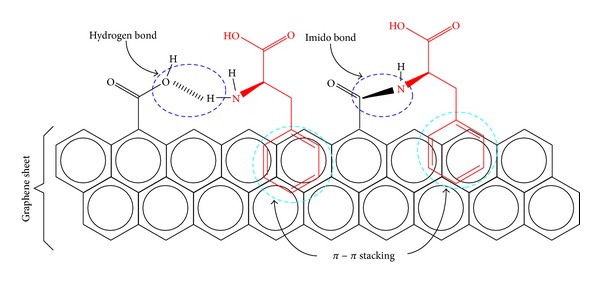
Illustration of possible mechanisms for Phe adsorption onto carbonaceous surfaces (adapted from Piao et al. [16]).

Several mechanisms have been proposed in the literature, and some of them were verified for the adsorption of phenylalanine onto carbonaceous and noncarbonaceous materials [10, 11, 16, 18]. Hydrophobic interactions of the *π*-*π* type are recognized to be the main type of interaction between the phenyl rings of the Phe molecules and graphene rings of the adsorbent surfaces [10]. However, other adsorption mechanisms were also demonstrated to occur, such as hydrogen bonding between Phe amino groups and oxygenated groups at the surface of the adsorbent and the formation of imido bonds between the Phe amino groups and carboxyl groups at the surface of the adsorbent (Figure 6). Recently, it was also demonstrated that, for adsorbents prepared by thermochemical activation with phosphoric acid at relatively low temperatures (*T* ≤ 450°C), phosphonates can be formed during adsorption by the interaction of carboxylic groups of Phe molecules with phosphate groups that link the graphene sheets comprising the main structure of the adsorbent [10]. Given the similarities in the molecular structure of Tyr and Phe, the adsorption mechanisms for Tyr are expected to be the same as those for Phe.

## 4. Conclusions

An adsorbent was prepared from corn cobs and used for the removal of tyrosine from both a single component aqueous solution and a binary solution with phenylalanine. For single component solutions, the adsorption behavior of tyrosine was similar to that of phenylalanine, with the prepared sorbent presenting a much lower adsorption capacity for Tyr (14 mg g^−1^) than for Phe (109 mg g^−1^). Adsorption kinetics for Tyr was satisfactorily described by a pseudosecond-order model, as it was also observed for Phe removal. In binary mixtures, the presence of Tyr in Phe solutions favored Phe adsorption, whereas the opposite behavior was observed for the presence of Phe in Tyr solutions, indicating that Phe will be removed preferably to Tyr by the prepared adsorbent. Thus, this adsorbent is a potential candidate for use in the removal of Phe from protein hydrolisates to be used in dietary formulations for phenylketonuria treatment, given that it will efficiently adsorb Phe without significant alterations in the amount of Tyr present in the hydrolisates.

## Figures and Tables

**Table 1:** Kinetic parameters for Tyr adsorption.

	Tyr initial concentration (mg L^−1^)				
	20	30	50	75	100
	25°C				

*q*_*e*_ (experimental)	2.01	2.74	4.63	7.31	9.11
Pseudofirst-order					
*k*_1_ (h^−1^)	6.212	3.754	1.735	1.772	1.786
*q*_*e*_ (estimated) (mg g^−1^)	1.76	2.52	4.52	7.20	9.02
*r* ^2^	0.881	0.897	0.912	0.955	0.955
RMS (%)	16.004	7.201	9.638	8.304	8.375
Pseudosecond-order					
*k*_2_ (g mg^−1^ h^−1^)	4.616	2.126	0.508	0.307	0.245
*q*_*e*_ (estimated) (mg g^−1^)	1.92	2.72	4.94	7.93	9.95
*r* ^2^	0.953	0.961	0.943	0.973	0.967
RMS (%)	3.990	4.410	7.511	5.881	6.449

	35°C				

*q*_*e*_ (experimental)	2.16	2.77	4.80	6.79	8.20
Pseudofirst-order					
*k*_1_ (h^−1^)	2.991	6.415	4.020	11.674	4.182
*q*_*e*_ (estimated) (mg g^−1^)	2.06	2.51	4.55	6.14	7.87
*r* ^2^	0.984	0.955	0.972	0.942	0.982
RMS (%)	7.705	3.573	3.111	3.543	3.377
Pseudosecond-order					
*k*_2_ (g mg^−1^ h^−1^)	1.791	3.375	1.068	2.601	0.720
*q*_*e*_ (estimated) (mg g^−1^)	2.26	2.72	4.50	6.60	8.52
*r* ^2^	0.993	0.986	0.982	0.983	0.999
RMS (%)	1.607	1.783	4.898	1.988	0.362

	45°C				

*q*_*e*_ (experimental)	1.95	2.62	4.55	6.44	8.12
Pseudofirst-order					
*k*_1_ (h^−1^)	3.831	5.204	3.701	6.713	4.456
*q*_*e*_ (estimated) (mg g^−1^)	1.80	2.53	4.555	5.94	7.59
*r* ^2^	0.970	0.991	0.964	0.940	0.921
RMS (%)	9.728	1.790	7.532	4.478	5.749
Pseudosecond-order					
*k*_2_ (g mg^−1^ h^−1^)	2.588	2.811	2.492	1.581	0.867
*q*_*e*_ (estimated) (mg g^−1^)	1.98	2.73	4.62	6.40	8.15
*r* ^2^	0.990	0.997	0.993	0.987	0.974
RMS (%)	2.569	1.345	1.588	2.147	2.994

**Table 2:** Pure Tyr equilibrium isotherm models and fitting parameters.

Model	Equation	Parameter values			*r* ^2^			RMS (%)		
		25°C	35°C	45°C	25°C	35°C	45°C	25°C	35°C	45°C
Langmuir	$q_{e}=\frac{q_{m}K_{L}C_{e}}{1+K_{L}C_{e}}$	$\begin{array}{c} K_{L}=0.164\\ q_{m}=14.19 \end{array}$	$\begin{array}{c} K_{L}=0.140\\ q_{m}=10.87 \end{array}$	$\begin{array}{c} K_{L}=0.106\\ q_{m}=11.33 \end{array}$	0.992	0.996	0.995	8.443	3.605	4.226

Freundlich	*q*_*e*_ = *K*_*F*_ *C*_*e*_^*n*^	$\begin{array}{c} K_{F}=2.398\\ n=1.777 \end{array}$	$\begin{array}{c} K_{F}=1.957\\ n=2.109 \end{array}$	$\begin{array}{c} K_{F}=1.619\\ n=1.922 \end{array}$	0.984	0.959	0.970	15.514	12.140	11.239

Temkin	*q*_*e*_ = (RT/*b*)ln⁡(*K*_*T*_ *C*_*e*_)	$\begin{array}{c} K_{T}=1.482\\ \text{RT}/b=1.422 \end{array}$	$\begin{array}{c} K_{T}=1.258\\ \text{RT}/b=1.080 \end{array}$	$\begin{array}{c} K_{T}=0.968\\ \text{RT}/b=1.124 \end{array}$	0.991	0.994	0.999	3.350	4.291	1.417

Langmuir-Freundlich	$\begin{array}{c} q_{e}=\frac{K_{LF}q_{m}C_{e}^{n}}{1+K_{LF}C_{e}^{n}} \end{array}$	$\begin{array}{c} K_{LF}=0.1672\\ q_{m}=10.41\\ n=1.527 \end{array}$	$\begin{array}{c} K_{LF}=0.133\\ q_{m}=9.78\\ n=1.181 \end{array}$	$\begin{array}{c} K_{LF}=0.103\\ q_{m}=10.1\\ n=1.144 \end{array}$	0.998	0.998	0.996	1.763	2.766	3.722

*q*_*e*_ (mg g^−1^) is the equilibrium adsorption capacity; *C*_*e*_ (mg L^−1^) is the solute concentration in the aqueous solution, after equilibrium; *q*_*m*_ (mg g^−1^) is the maximum adsorption capacity; the remaining constants are empirical parameters associated to each specific model.

**Table 3:** Binary systems equilibrium isotherm models and fitting parameters.

Model	Primary amino acid—interfering amino acid	*q*_*m*_ (mg g^−1^)	*K*_1_/*K*_2_*	RMS (%)	*r* ^2^
Langmuir					
(a) Phe removal	Phe-Tyr	78.46	8.3	3.194	0.996
(b) Tyr removal	Tyr-Phe	6.78	0.1	0.505	0.999
Freundlich					
(c) Phe removal	Phe-Tyr	79.17	7.7	3.488	0.995
(d) Tyr removal	Tyr-Phe	6.35	0.2	0.359	0.999

*Subscripts 1 and 2 refer to the amino acid being removed (1) and the interferent (2).
